# Confronting the Crisis: Actions to Address Maternal Morbidity and Mortality Among Black Women in Rural Georgia

**DOI:** 10.5888/pcd22.250125

**Published:** 2025-07-10

**Authors:** Jalen Robinson, Jaliyah Screen, Carey Roth Bayer

**Affiliations:** 1 Morehouse School of Medicine, Atlanta, Georgia

Maternal mortality remains a public health crisis in the US, disproportionately affecting Black women, who face substantially higher risks compared with their White counterparts (1–3). In 2021, the national maternal mortality rate was 32.9 deaths per 100,000 live births (1). However, the rate among non-Hispanic Black women was 69.9 per 100,000 live births — nearly 2.6 times higher than of the rate among non-Hispanic White women (1). In Georgia, the maternal mortality rate is 66.3 per 100,000 live births, the second highest rate in the US (2). Women in rural areas (ie, <50,000 people) face maternal mortality risks up to 50% higher than their urban counterparts; of Georgia’s 159 counties, 120 are classified as rural (4). The disparities are even more pronounced among Black women in rural Georgia, whose maternal mortality rate is double that of their rural White counterparts and 30% higher than that among Black women in urban areas (4). These statistics underscore the urgent need for focused, targeted interventions to address maternal mortality among Black women, particularly in the rural communities of Georgia.

## Why Is the Maternal Morbidity and Mortality Health Crisis a Challenge in Rural Georgia?

Rural Georgia faces substantial health care infrastructure challenges, including a shortage of hospitals and specialized maternal care providers (4). Economic barriers, including high poverty rates, further limit women’s ability to access transportation, resulting in delayed or missed health care appointments that often affect maternal health (3). Cultural and language barriers also persist, as many health care providers lack the cultural competency needed to effectively engage with Black women, which discourages them from seeking care (3). High rates of postpartum depression and anxiety worsen maternal morbidity and mortality, as limited mental health resources leave many women in medically underserved areas to struggle in silence, which affects both maternal and infant health (5). Additionally, the shortage of health care providers, coupled with the high turnover rate of providers in rural communities, disrupts continuity of care, making it difficult for women to establish long-term, trusting relationships with their health care providers (4).

## Existing and Historical Policy Shortcomings

### US federal policies

Historically, federal programs like the Maternal and Child Health Services Block Grant, the National Health Service Corps, and the Healthy Start program have been designed to improve maternal health outcomes, especially for underserved populations (4,6). However, these policies have fallen short of addressing the needs of Black women, particularly in rural areas. Despite the intent, underfunding and inconsistent implementation have limited their effectiveness. The National Health Service Corps, which offers loan repayment incentives for health care providers to work in medically underserved areas, has struggled with high turnover rates in rural regions, preventing continuity of care (4,5). The Healthy Start program, meant to provide services like home visits and early screenings, often fails to reach remote communities due to geographic barriers and resource limitations (4).

### State of Georgia policies

Medicaid expansion, shown to reduce maternal mortality rates in states that adopted it, would have provided low-income women with critical prenatal and postpartum care. However, Georgia’s decision not to expand Medicaid leaves many women without health insurance, forcing them to forgo necessary care. The state’s lack of culturally competent care and a shortage of health care professionals, especially in rural areas, continue to perpetuate disparities.

Furthermore, Georgia’s 6-week abortion ban has substantially affected the Black maternal health crisis by exacerbating existing disparities and limiting reproductive choices. The ban restricts access to abortion services for Black women, who already face systemic barriers to comprehensive health care. This restriction forces some women to carry unwanted pregnancies to term, increasing the risk of maternal complications and adverse health outcomes. The ban also imposes additional financial and emotional burdens, particularly on those in rural areas with limited resources. By reducing access to safe and timely reproductive care, the policy further deepens the challenges.

## Proposed Solutions and Innovative Strategies

Solutions for addressing the challenges exist at many levels. We suggest ways to link solution ideas to levels of leadership (Figure).

**Figure:**
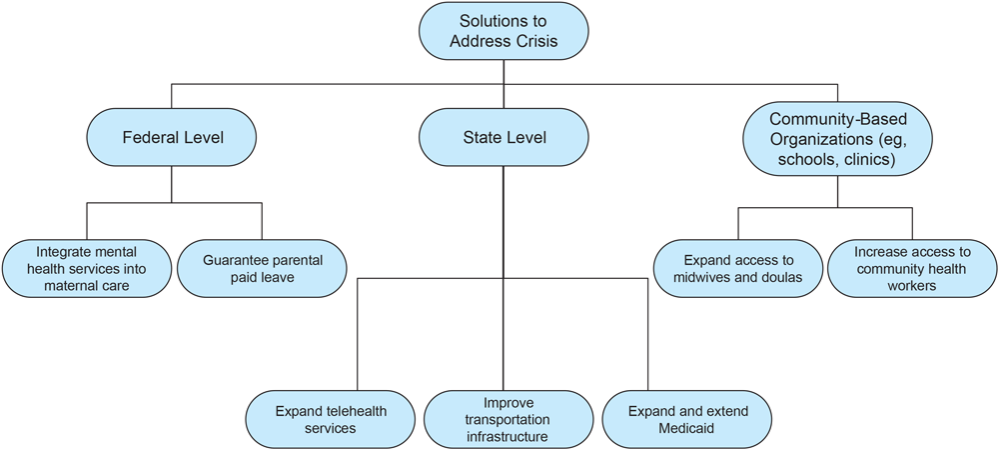
Hierarchy of leadership levels and solutions to address existing challenges in maternal health among Black women in rural Georgia.

### Community-based organizations

To address the disparities in Black maternal health, expanding access to midwives and doulas is essential. Midwifery care reduces preterm births and cesarean delivery rates and improves breastfeeding outcomes, particularly among Black mothers. Doulas provide invaluable emotional and physical support during pregnancy and childbirth, mitigating racial biases and fostering trust in the health care system (3). Programs like Morehouse School of Medicine’s rural doula initiative have demonstrated effectiveness in improving maternal health outcomes in Georgia and could serve as a model for expansion statewide. Additionally, implementing policies that streamline doula reimbursement and simplify administrative paperwork is crucial to removing barriers, supporting doula workforce sustainability, and enhancing access to these vital services (3).

Increasing the availability of community health workers (CHWs) is also a vital strategy. CHWs play a pivotal role in bridging gaps between medically underserved populations and health care providers, offering culturally competent care; guiding patients through the health care system; providing prenatal education; and assisting with transportation, which can be challenging in rural areas where long travel distances often delay care (7). Strengthening CHW programs would help ensure that Black women in rural Georgia receive the necessary support to improve maternal health outcomes.

### Solutions at the state level

Telehealth offers a promising solution for enhancing access to maternal care in rural Georgia. Virtual consultations, remote monitoring, and postpartum care provide timely access to specialists, especially in areas lacking obstetricians and family physicians (8). However, for telehealth to be effective, substantial investment in infrastructure is needed, including expanding broadband and high-speed internet access to underserved rural areas.

Expanding Medicaid would also provide access to comprehensive health care coverage for many low-income women, helping to reduce pregnancy-related complications and mortality rates (6). Additionally, extending Medicaid postpartum coverage to one year is crucial for addressing mental health and chronic conditions that contribute to maternal mortality rates (3,6). Evidence from other states that have expanded Medicaid shows marked improvements in maternal health outcomes.

Improving transportation infrastructure and implementing mobile health clinics are crucial to addressing the maternal mortality crisis in rural Georgia. Many rural counties lack adequate hospital facilities, requiring women to travel long distances — often more than 50 miles — to access essential care (4). Expanding public transit options, offering shuttle programs, or partnering with ride-share services can help alleviate these logistical barriers. Mobile health clinics can further reduce access gaps by delivering essential maternal health services, such as prenatal checkups, screenings, and education, directly to medically underserved areas, ensuring women receive the care they need promptly (9).

### Solutions at the federal level

Integrating mental health services into maternal care is critical to addressing the mental health challenges faced by Black women in rural Georgia. By embedding mental health screenings and treatment within prenatal and postpartum care, the health care system can better address these issues (5). Expanding Medicaid to cover mental health services would ensure that Black women have access to comprehensive care, improving both their physical and mental health before, during, and after pregnancy.

Guaranteeing paid leave to new parents is a critical policy change, particularly for Black women, who are disproportionately affected by a lack of paid leave options. Currently, only 30% of Black rural women have access to paid leave (10). Implementing paid leave would allow women to recover from childbirth and care for their families without risking their financial security. Because many Black mothers serve as the primary breadwinners in their households, paid leave would improve maternal and child health outcomes by allowing women the time they need for postpartum recovery, ultimately supporting their health and economic stability (10).

## Who Would Benefit From These Solutions?

These innovative solutions are designed to bring substantial improvements to Black women in rural Georgia, who disproportionately face challenges related to maternal health. Expanding access to midwifery care, doulas, and CHWs would provide these women with personalized, culturally competent care at every stage of pregnancy, during childbirth, and postpartum. This comprehensive support ensures that Black women receive the attention and resources they need in a manner that is sensitive to their unique cultural and health needs. Additionally, rural health care providers stand to benefit from enhanced support for maternal health professionals. By addressing existing provider shortages and improving health care access, we can reduce the strain on the system and enhance job satisfaction among providers, ultimately leading to better outcomes for all involved.

## Future Outlook

If effectively implemented, these innovative approaches could transform maternal health care in rural Georgia, reducing maternal mortality and morbidity while strengthening the state’s health care system and fostering greater equity and inclusivity. A more integrated and comprehensive health care system would guarantee that women, particularly Black women in rural areas, have access to continuous, culturally competent care throughout pregnancy, childbirth, and the postpartum period. Moreover, this approach could serve as a framework for other states facing similar challenges, promoting nationwide change and improving maternal health outcomes across the country.
